# Unbiased comparison and modularization identify time-related transcriptomic reprogramming in exercised rat cartilage: Integrated data mining and experimental validation

**DOI:** 10.3389/fphys.2022.974266

**Published:** 2022-09-15

**Authors:** Jiarui Cui, Yo Shibata, Keiji Itaka, Jun Zhou, Jiaming Zhang

**Affiliations:** ^1^ School of Rehabilitation and Health Preservation, Chengdu University of Traditional Chinese Medicine, Chengdu, China; ^2^ Department of Conservative Dentistry, Division of Biomaterials and Engineering, Showa University School of Dentistry, Tokyo, Japan; ^3^ Department of Biofunction Research, Institute of Biomaterials and Bioengineering, Tokyo Medical and Dental University (TMDU), Tokyo, Japan; ^4^ Department of Orthopedics, Tongji Hospital, Tongji Medical College, Huazhong University of Science and Technology, Wuhan, China

**Keywords:** osteoarthritis, cartilage, transcriptome, exercise, exercise time

## Abstract

Exercise is indispensable for maintaining cartilage integrity in healthy joints and remains a recommendation for knee osteoarthritis. Although the effects of exercise on cartilage have been implied, the detailed mechanisms, such as the effect of exercise time which is important for exercise prescription, remain elusive. In this study, bioinformatic analyses, including unbiased comparisons and modularization, were performed on the transcriptomic data of rat cartilage to identify the time-related genes and signaling pathways. We found that exercise had a notable effect on cartilage transcriptome. Exercise prominently suppressed the genes related to cell division, hypertrophy, catabolism, inflammation, and immune response. The downregulated genes were more prominent and stable over time than the upregulated genes. Although exercise time did not prominently contribute to the effects of exercise, it was a factor related to a batch of cellular functions and signaling pathways, such as extracellular matrix (ECM) homeostasis and cellular response to growth factors and stress. Two clusters of genes, including early and late response genes, were identified according to the expression pattern over time. ECM organization, BMP signaling, and PI3K-Akt signaling were early responsive in the exercise duration. Moreover, time-related signaling pathways, such as inositol phosphate metabolism, nicotinate/nicotinamide metabolism, cell cycle, and Fc epsilon RI signaling pathway, were identified by unbiased mapping and polarization of the highly time-correlated genes. Immunohistochemistry staining showed that *Egfr* was a late response gene that increased on day 15 of exercise. This study elucidated time-related transcriptomic reprogramming induced by exercise in cartilage, advancing the understanding of cartilage homeostasis.

## Introduction

Osteoarthritis (OA) is the most common degenerative joint disease and a leading cause of disability and chronic pain (Neogi, 2013). OA is a whole joint disease involving all joint tissues (i.e., cartilage, synovial membrane, menisci, ligaments, and infrapatellar fat pad), characterized by degeneration and inflammation of the affected tissues (Clockaerts et al., 2010; Englund et al., 2012; Poole, 2012; Glyn-Jones et al., 2015; Marshall et al., 2018; Sanchez-Lopez et al., 2022). OA impacts approximately 1 in 3 adults over the age 60 of years and causes a significant social-economic burden (Felson, 2010; Zhang et al., 2019). Pharmacological treatments of OA involve the use of non-steroidal anti-inflammatory drugs, opioid or non-opioid analgesics, and intra-articular injections of steroids and hyaluronic acid, among which oral medications may have significant negative gastrointestinal side effects (Glyn-Jones et al., 2015; Pedersen and Saltin, 2015). In addition to the pharmacological treatments and joint replacement surgery, life style modifications, regular exercise, and physical therapy are widely recommended in OA management, which attenuates symptoms and improves joint function and/or quality of life (Zhang et al., 2008; Schiphof et al., 2018; Katz et al., 2021).

Exercise is indispensable for maintaining cartilage integrity in healthy joints and remains a core recommendation for knee OA, including anaerobic, aerobic, flexibility workouts, and aquatic exercise, inducing the benefits on knee-related and health-related outcomes, such as pain release and joint function and life quality improvements (Hunter and Eckstein, 2009; Musumeci et al., 2015; Collins et al., 2019). Since excessive loading aggravates the pathological condition of OA, it is essential to understand the effects of exercise and propose an evidence-based protocol in which the type, duration, and intensity are assumed to induce joint improvement without disease aggravation (Musumeci et al., 2014; Gardner et al., 2017). In the perspective of precision medicine, exercise should be prescribed in the same way as pharmacological treatment, deciding on the “dosage” and “formulation” that are suitable for individuals (Swisher, 2010). Some findings have demonstrated partially the mechanisms of exercise (Blazek et al., 2016). However, the knowledge gap of the molecular mechanisms relevant to exercise has limited us to exploit the therapeutic potential of exercise and maximize its effectiveness. It is necessary to explore the effects of different exercise delivery (exercise dose, frequency, methods, etc.) on the joint system and their detailed mechanisms to facilitate a beneficial “exercise prescription”.

Here, we performed integrated bioinformatic analysis to identify the genes and signaling pathways related to exercise effects on healthy cartilage. Moreover, we focused on the effects of exercise time on transcriptomic reprogramming and the candidate genes or signaling pathways that may contribute to this reprogramming. This study provides a comprehensive understanding of the effects of exercise dosage on cartilage transcriptome.

## Materials and methods

### Data collection and pre-processing

The dataset was obtained from the National Center For Biotechnology Information (NCBI) Gene Expression Omnibus (GEO) with the accessing number GSE74898 on the platform of GPL6247 (Affymetrix Rat Gene 1.0 ST Array), which was designed to investigate the effects of exercise (low intensity treadmill walking, 12 m/min for 45 min daily) or exercise time (0, 2, 5, or 15 days) on healthy Sprague-Dawley rats (12–14 weeks old females) (Blazek et al., 2016). The dataset contains 12 articular cartilage samples in total. Twelve samples were assigned into 4 groups according the exercise time, including the control group (D0, *n* = 3) and exercised groups (D2, *n* = 3; D5, *n* = 3; D15, *n* = 3). Background correction and quantile normalization were performed by robust multi-array analysis (RMA) (Joerger et al., 2014). Averages were considered as gene expression values if the multiple probes were mapped to the same gene symbol. Batch effect was carefully considered by the package by *R* package BatchI (Papiez et al., 2019). After these processes, the gene expression matrix was obtained for further analysis. Sample relationships was estimated by principal component analysis (Zhou et al., 2022).

### Transcriptomic comparison, persistent gene identification, and pathway enrichment

To investigate the gene-level difference between two groups, differentially expressed genes (DEGs) were identified using limma method by the threshold set as false discovery rate (FDR) < 0.05 (Ritchie et al., 2015). Persistent genes were identified by overlapping the DEGs between D0 and D2, D5, or D15. Persistence scores were estimated by the RRA algorithm (Kolde et al., 2012; Zhou et al., 2022) to integrate the ranks of fold-change values in different comparisons. A high persistence score indicates a stable change of gene expression to exercise. DEGs were subjected to the pathway enrichment analysis using over-representation analysis (Subramanian et al., 2005). Gene Ontology (GO) and the Kyoto Encyclopedia of Genes and Genomes (KEGG) analyses were performed by the *R* package clusterProfiler (v3.11) (Cheng et al., 2021; Wu et al., 2021). ClusterProfiler is an *R* package for comparing biological themes among gene clusters and functionally annotates the specific gene sets (Yu et al., 2012). The functions enrichGO and enrichKEGG enveloped in this *R* package were utilized to perform enrichment test for GO terms and KEGG pathways based on hypergeometric distribution, the results of which were visualized by bubble plot (Tang et al., 2018). The heatmap of enriched genes involved in the significant pathways was plotted by function heatplot.

### Gene modularization and gene-time correlation estimation

Weighted correlation network analysis (WGCNA) was utilized in gene modularization. The scale-free gene network was built up based on gene co-expression by *R* package WGCNA (Langfelder and Horvath, 2008). Firstly, the function softConnectivity and pickSoftThreshold enveloped in WGCNA were utilized to select the appropriate soft-thresholding power to construct network by calculating the scale-free topology fit index for different powers and balance the mean connectivity and scale independence. If the scale-free topology fit index values above 0.9 for low powers (<30), it means that the topology of the network is scale-free (Liu et al., 2017). After scale-free processing, adjacency matrix was obtained and further converted into Topological Overlap Matrix which reflects the interrelationship of genes taking consideration of not only the direct but also the indirect co-relations. In this matrix, parts of genes have high topological overlap with others, which represents they can be classified into a biologically meaningful module. Secondly, the modules were identified by Dynamic Tree Cut algorithm by the parameters set up as minModuleSize = 30 and deepsplit = 2. Based on the module eigengenes which were calculated to represent the modules in a one-dimensional vector, the close modules are merged by hierarchical cluster analysis and Merged Dynamic algorithm (cutheight = 0.25) (Chen et al., 2018). Finally, Pearson Correlation was employed to quantify the correlation between module eigengenes and phenotypes. By the threshold of the Pearson Correlation Coefficient (|r| > 0.4 and *p* < 0.05), time-related gene modules were determined. In a time-related module, the genes with |Module Membership (MM)| > 0.7 and Gene Significance (GS)| > 0.7 were defined as the high time-correlated genes (Li et al., 2020).

### Gene polarization and time-related pathway identification

Total genes involved in WGCNA were ranked by the absolution value of time correlation to generate a pre-ranked gene list. The pre-ranked gene list was challenged by multiple KEGG pathway signatures using Gene Set Enrichment Analysis (GSEA). The function gseKEGG enveloped in clusterProfiler was employed to identify the time-relevant signaling pathways (Subramanian et al., 2005). The original time correlations of leading-edge gene set were visualized by function heatplot.

### Rat exercise model and candidate gene validation

All animal experimental protocols were approved by Tongji Medical College, Huazhong University of Science and Technology (Wuhan, China). To validate candidate time-responsive genes, we performed the exercise model as Blazek et al. (2016) designed (low intensity treadmill walking, 12 m/min for 45 min daily). A total of 15 male Sprague-Dawley rats (12 weeks old females) were assigned into three groups according to exercise time: Day 0, Day5, Day 15. Each group contained 5 animals. The animals were maintained on a 12 h light/dark cycle under constant temperature (22°C ± 1°C) and were able to move freely in the cages and had free access to autoclaved food and water. We harvested the knee joints on day 0, day 5, and day 15. After fixed in 4% paraformaldehyde solution for 48 h at room temperature, the knee joints were decalcified by 10% EDTA solution at 4°C, dehydrated in graded ethanol solutions, and embedded in paraffin. The tissues were cut into 4 µm sagittal sections. Immunohistochemistry staining (IHC) with EGFR antibody (66455-1-Ig, Proteintech, United States) was performed and quantically analyzed as the previous publication described (Wang et al., 2022).

### Statistical analysis

One-way ANOVA statistics method was used for comparison among multiple groups. All data was displayed as means ± SD and analyzed using GraphPad Prism software version 7.0. *p* < 0.05 was considered statistically significant.

## Results

### Exercise notably remodels cartilage transcriptome

**FIGURE 1:**
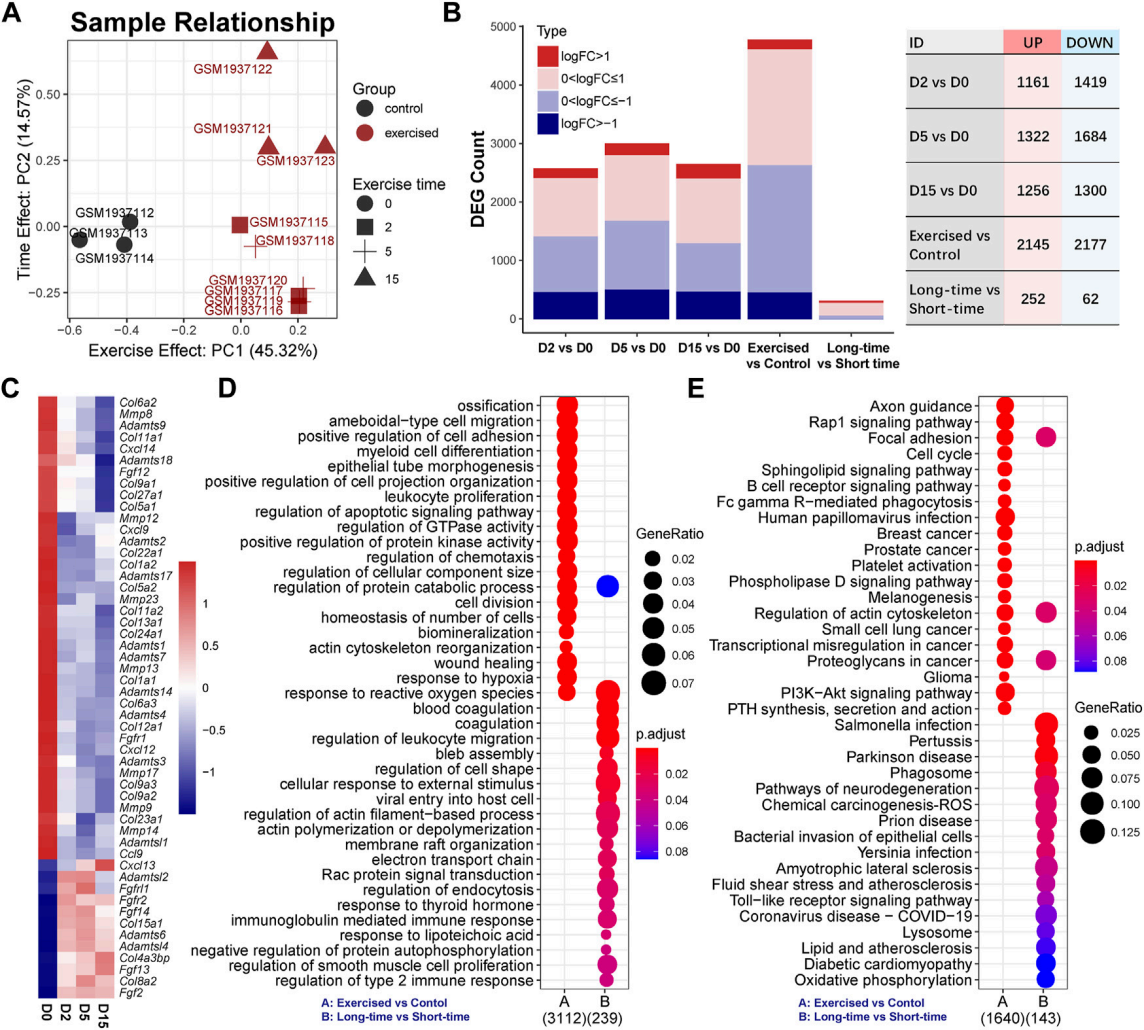
Exercise has a notable effect on the cartilage transcriptome. **(A)** Sample relationship estimated by principal component analysis. PC1, the first principal component; PC2, the second principal component. **(B)** The numbers of differentially expressed genes (DEGs) in serial comparisons. FC, fold-change value; DEG, differentially expressed gene. **(C)** The expression of collagens (COLs), matrix metallopeptidases (MMPs), tissue inhibitors of matrix metalloproteinases (TIMPs), a disintegrin and metalloproteinase with thrombospondin motifs (ADAMTS) family, C-X-C motif chemokine ligands (CXCLs), chemokine (C-C motif) ligands (CCLs), and fibroblast growth factors (FGFs) and receptors (FGFRs). **(D)** GO analysis and **(E)** KEGG pathway analysis of DEGs between the exercised and control groups or between the long-time and short-time groups. GeneRatio indicates the gene number ration in each GO function and KEGG pathway. The color and size of dot represent *p*-value adjusted by Benjamini and Hochberg method and gene number assigned to the corresponding GO function and KEGG pathway, respectively.

To investigate the effect of exercise on transcriptome, we employed a dimensionality reduction method to characterize the overall difference between the exercised and control groups. As the PCA result indicated (Figure 1A), the exercised samples (*n* = 9) were clearly separated from the controls (*n* = 3). Of note, the first principal component (PC1) that indicates the main difference between samples, accounted for 45.31%, while the second component (PC2) only accounted for 14.57%, of the overall difference. The result suggests that the main difference between these samples on transcriptome was in relation to exercise, not the exercise time. The D15 samples were separated from the D2 and D5 samples while the D2 and D5 samples were overlapped. This suggests that, although the transcriptome difference among the samples of individual exercise time was small, the difference could be characterized between D15 and the shorter time than D15.

The DEG numbers of serial comparisons were in line with the PCA result. As shown in Figure 1B, the DEG number of the individual time points compared with the control were comparable and the suppressed genes were more prominent than the activated genes. There were no positive hits between individual time points that met the criteria of FDR < 0.05 (Supplementary Table S1). Since FDR is estimated by multiple tests and very strict for a limited sample size, we pooled the D0 and D5 as a group, named the short-time group, and redefined the D15 as the long-time group. Although the DEG number between the long-time and short-time groups, as expected, was small, 314 genes were responsive to exercise time (Figure 1B), including 252 upregulated and 62 downregulated genes. Of these genes, only ∼15.92% (*n* = 50) overlapped with the DEGs (*n* = 4,322) between the exercised and control groups (Supplementary Figure S1).

To explore the effect of exercise, we first focused on the comparison of the exercised and control groups and investigated several groups of genes that are known critical for chondrocytes, including collagens (COLs), matrix metallopeptidases (MMPs), tissue inhibitors of matrix metalloproteinases (TIMPs), a disintegrin and metalloproteinase with thrombospondin motifs (ADAMTS) family, C-X-C motif chemokine ligands (CXCLs), chemokine (C-C motif) ligands (CCLs), and fibroblast growth factors (FGFs) and receptors (FGFRs) (Figure 1C). *Col2a1* expression was not changed by exercise (Supplementary Table S2). Hypertrophy markers, *Col1a1* and *Col1a2* were reduced by exercise (Figure 1C) while *Col10a1* was not changed (Supplemental Table 2). Moreover, a batch of collagens was suppressed by exercise. Two critical MMPs related to OA, *Mmp9* and *Mmp13*, were suppressed by exercise in a time-dependent manner. MMP inhibitors *Timp2* and *Timp4* were increased. Most ADAMTS genes were also suppressed except for *Adamts6*, *Adamtsl2*, and *Adamtsl4* which were increased. Among CXCLs and CCLs, *Cxcl9*, *Cxcl12*, *Cxcl14*, and *Ccl9* were decreased while *Cxcl13* was notably increased in a clear time-dependent manner. Three FGFs were significantly elevated by exercise, including *Fgf2*, *Fgf13*, and *Fgf14*. Of note, *Fgf2* showed a consistent increase independent of time. These alternations of gene expression indicate a notable change in chondrocyte function under exercise stimulation. Some genes showed a time-dependent response to exercise.

Functional annotations of the exercise-responsive genes (exercised vs. control; *n* = 4,322) and time-related genes (long-time vs. short-time; *n* = 314) showed the biological processes and signaling pathways they involve (Figures 1D,E). GO analysis showed that chondrocyte behaviors (division, migration, and adhesion) and function (ossification, biomineralization, metabolism) were related to exercise, while the most notable biological process was the response to reactive oxygen species (ROS) (Figure 1D). KEGG analysis showed a batch of signaling pathways. Of note, Rap1 and PI3K-Akt signaling pathways were significantly affected (Figure 1E). Moreover, lysosome and phagosome were significantly related to exercise time. A group of genes related to oxidative phosphorylation indicated the changes in mitochondrial function and energy metabolism related to exercise time (Figure 1E).

### Persistent responsive genes are related to cell division, inflammation, and metabolism

**FIGURE 2:**
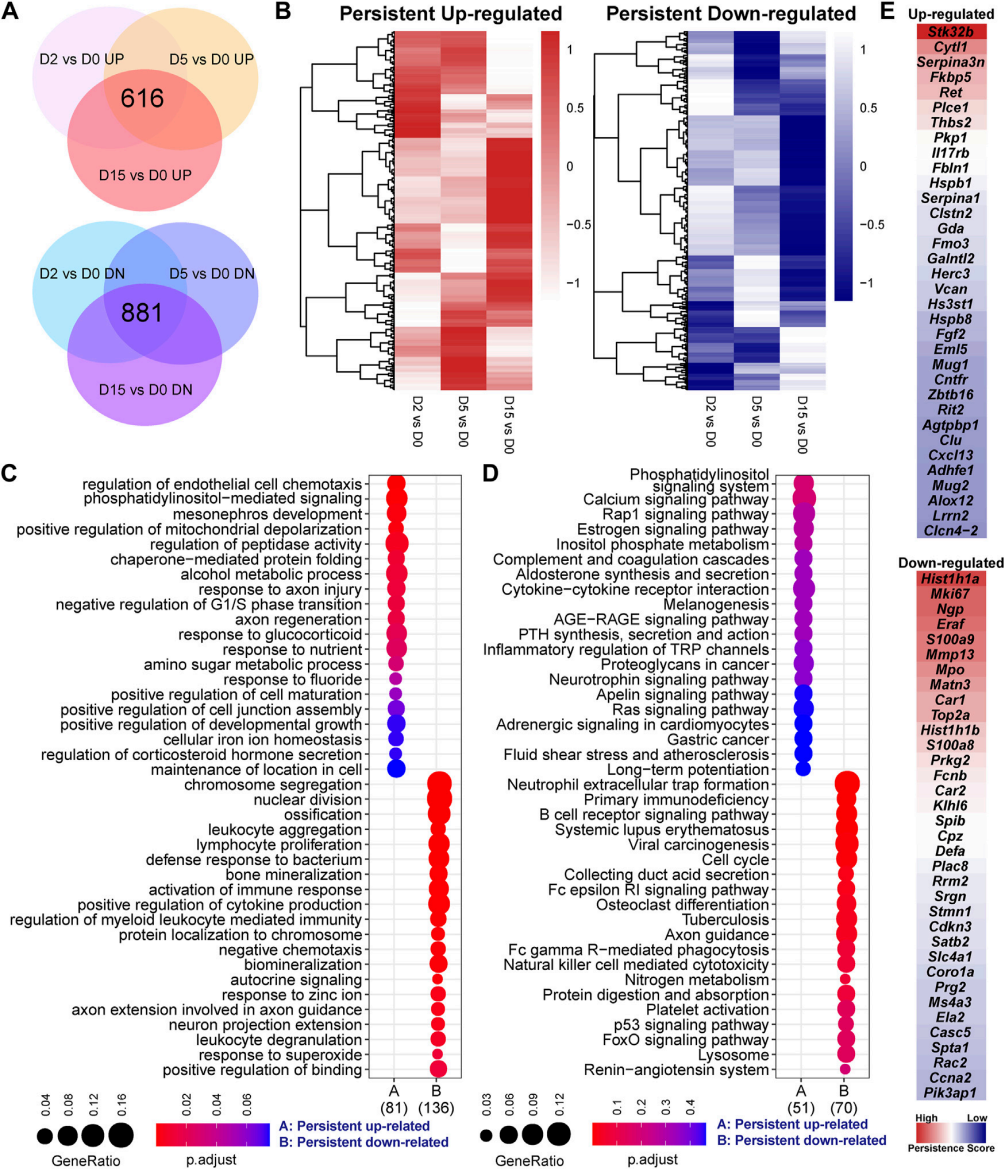
Persistent responsive genes are related to cell division, inflammation, and metabolism. **(A)** Overlapped genes of three set of differentially expressed genes of serial comparisons, including 616 persistent upregulated and 881 persistent downregulated genes. **(B)** Heatmaps of the fold-change values of two clusters of persistent genes. The heatmap patterns show that some genes exhibit a time-related change of gene expression. **(C)** GO analysis and **(D)** KEGG pathway analysis of persistent genes. GeneRatio indicates the gene number ration in each GO function and KEGG pathway. The color and size of dot represent *p*-value adjusted by Benjamini and Hochberg method and gene number assigned to the corresponding GO function and KEGG pathway, respectively. **(E)** The top 35 up-regulated or downregulated genes based on persistence score. A high persistence score indicates a stable response in the exercise duration.

By overlapping three set of DEGs individually, we found that a large portion of genes were consistently increased or decreased by exercise in each time point (Figure 2A). Of note, the heatmap of fold-change values showed that, although these genes were persistently changed, some exhibited a time-related change of gene expression (Figure 2B). GO analysis characterized the biological processes related to these persistent responsive genes (Figure 2C). It is not surprising that some hits were similar with the result shown in Figures 1D,E. More importantly, the persistent upregulated genes were enriched into the cell chemotaxis, phosphatidylinositol-mediated signaling, mitochondrial depolarization, and peptidase activity, while cell division, ossification, bone mineralization, immune response, and cytokine production were related to the down-regulated genes. Of note, the up-regulated genes related to positive regulation of peptidase activity included *Alox12*, *Egln3*, *Fbln1*, *Gsn*, and *Ret*, while *LOC297568*, *Mug1*, *Mug2*, *Serpina1*, and *Serpina3n* are the negative regulators of this process (Supplementary Table S3). Moreover, the gene related to cell division and proliferation, such as *Ccnb1*, *Ccne1*, *Ccnf*, *Cdca3*, and *Mki67*, were consistently downregulated by exercise (Supplementary Table S4), suggesting that exercise stress suppresses cell division. The downregulated genes related to cytokine production included *C3*, *Camp*, *Cd84*, *Fcnb*, *Hmgb2*, *Laptm5*, *Mmp8*, *Panx3*, *Prg2*, *Ptprc*, *Sema7a*, *Spn*, *Syk*, and *Tyrobp* (Supplementary Table S4). KEGG showed the signaling pathways related to the persistent genes (Figure 2D). The persistent upregulated genes were related to the phosphatidylinositol signaling system, calcium signaling pathway, and Rap1 signaling pathway. Ten persistent downregulated gene showed a prominent enrichment in the neutrophil extracellular trap formation, including *C3*, *Camp*, *Ctsg*, *Hist1h2ao*, *Hist1h2bl*, *Hist2h3c2*, *Hist2h4*, *Mpo*, *Rac2*, and *Syk* (Supplementary Table S4).

To identify the genes that are persistently and stably affected by exercise, we defined a persistence score estimated by the RRA algorithm to integrate the ranks of fold-change values in different comparisons. As shown in Figure 2E, the downregulated genes were more prominent than the upregulated genes. The top five upregulated genes included *Stk32b*, *Cytl1*, *Serpina3n*, *Fkbp5*, *Ret*, *Plce1*, *Thbs2*, *Pkp1*, *Il17rb*, and *Fbln1*. The top five downregulated genes included *Hist1h1a*, *Mki67*, *Ngp*, *Eraf*, *S100a9*, *Mmp13*, *Mpo*, *Matn3*, *Car1*, and *Top2a*. Again, some persistent genes, such as *Mmp13* (Figure 1C), showed a time-related change of expression. However, the comparisons between the exercised and control groups or between the different time points were not sufficient to characterize the time-related transcriptomic response. The time-related changes might be small and not uniform. In the context of small sample size, these small changes may not meet the criteria of differential genes expression analysis in the context of a small sample size.

### Early and late responsive genes are identified by modularization of gene co-expression network

**FIGURE 3:**
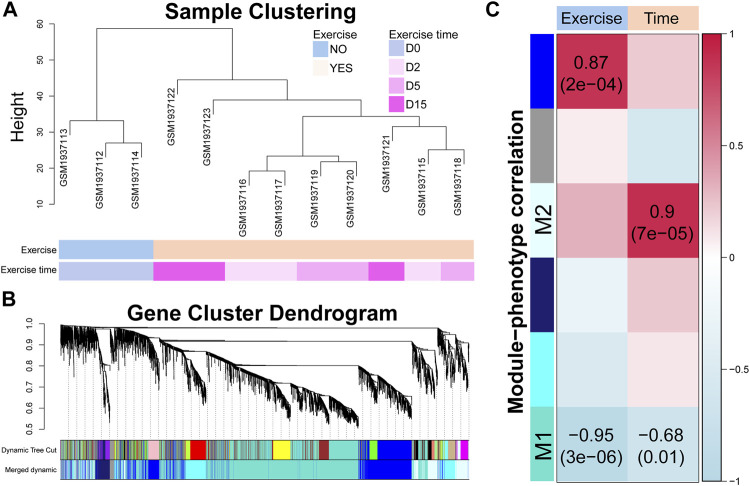
Time-correlated gene modules are identified by modularization method. **(A)** Sample clustering dendrogram based on the Euclidean distance. **(B)** Gene cluster dendrogram calculated by average linkage hierarchical clustering. The color row underneath the dendrogram shows the assigned original modules and the merged modules. **(C)** Correlation between module eigengene and exercise or exercise time. The value in each square and the *p*-value in parentheses reflect the correlation coefficient. *p* < 0.05 is considered statistically significant.

To identify the time-related transcriptomic response, we modularized genes and estimated the correlations between gene expression and exercise time. As shown in Figure 3A, the exercised samples were clustered as an individual branch while the samples of the different time points did not show a clear branch relationship. A total of 18 modules were identified by average linkage hierarchical clustering of gene expression and dynamic tree cut method, which were merged into six modules by module eigengene clustering (Figure 3B). The correlations between the eigengene of each module and exercise or exercise time (Figure 3C) showed that two modules (M1 and M2) were notably related to exercise time and the M2 module was also highly related to exercise. Although the eigengene of the module showed a strong correlation with exercise time, only a portion of genes was highly correlated with time (Supplementary Table S5). We used a criterion (|MM| > 0.7 and |GS| > 0.7) to select the high time-correlated genes for GO and KEGG analyses.

**FIGURE 4:**
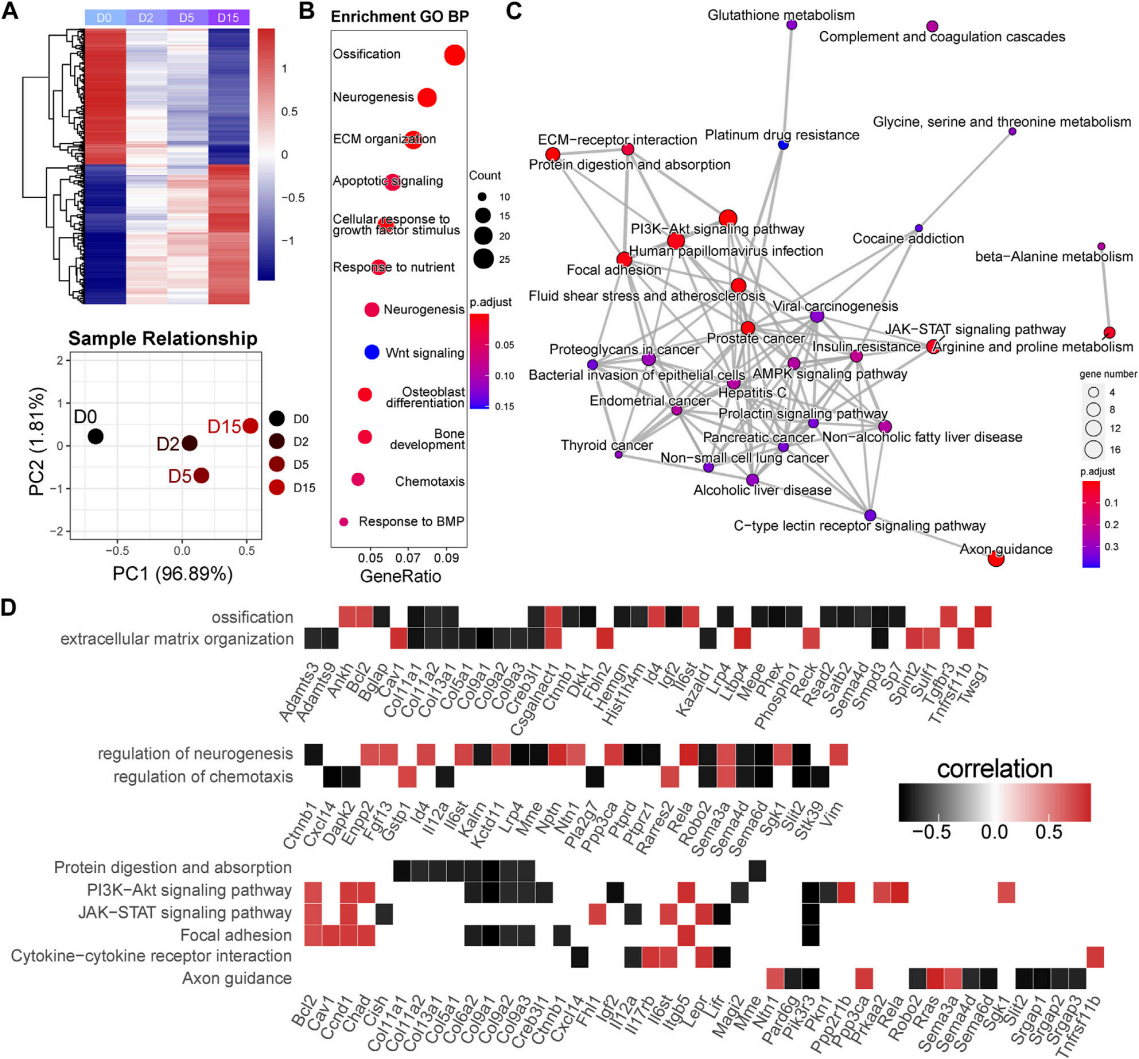
Early responsive genes are related to the critical events and signaling pathways for cartilage homeostasis. **(A)** Heatmap and principal component analysis of early responsive genes (*n* = 2,477). A clear difference between D0 and D2 or D5. PC1, the first principal component; PC2, the second principal component. **(B)** GO analysis of early responsive genes. GeneRatio indicates the gene number ration in each GO function. The color and size of dot represent *p*-value adjusted by Benjamini and Hochberg method and gene number assigned to the corresponding GO function, respectively. **(C)** Pathway network generated by KEGG analysis of early responsive genes. The color and size of dot represent *p*-value adjusted by Benjamini and Hochberg method and gene number assigned to the corresponding GO function, respectively. **(D)** Time correlation of genes in the enriched GO function and KEGG pathways.

In the M1 module, 2,477 genes were included. The heatmap of gene expression and PCA result showed a clear difference between D0 and D2 or D5 (Figure 4A). Based on this, we classified these genes as the early responsive genes in exercise duration. GO analysis showed that they were enriched in the biological processes highly related to chondrocyte functions, such as ossification, extracellular matrix (ECM), apoptosis, and response to BMP (Figure 4B). *Bglap*, *Bmper*, *Cav1*, *Dkk1*, *Sfrp5*, *Smpd3*, *Spint2*, *Sulf1*, *Tgfbr3*, and *Twsg1* were enriched in the BMP signaling (Supplementary Table S6). KEGG analysis showed that they were related to the PI3K-Akt signaling pathway, Focal adhesion, and JAK-STAT signaling pathway (Figure 4C). To clarify the key genes in these pathways, we plotted the correlations of the enriched genes with exercise time (Figure 4D). Interestingly, a batch of genes related to osteoblast differentiation, such as *Bglap*, *Sp7*, *Mepe*, and *Phex*, showed a strong negative correlation with exercise time. Of note, as the target gene of the Wnt signaling, decreased *Dkk1* suggested the suppression of Wnt signaling pathway. Moreover, *Tnfrsf11b*, an inhibitor of RANKL/RANK axis, was positively correlated with time. In the PI3K-Akt signaling, *Ppp2r1b*, *Prkaa2*, and *Sgk1*, also showed a positive correlation with time.

**FIGURE 5:**
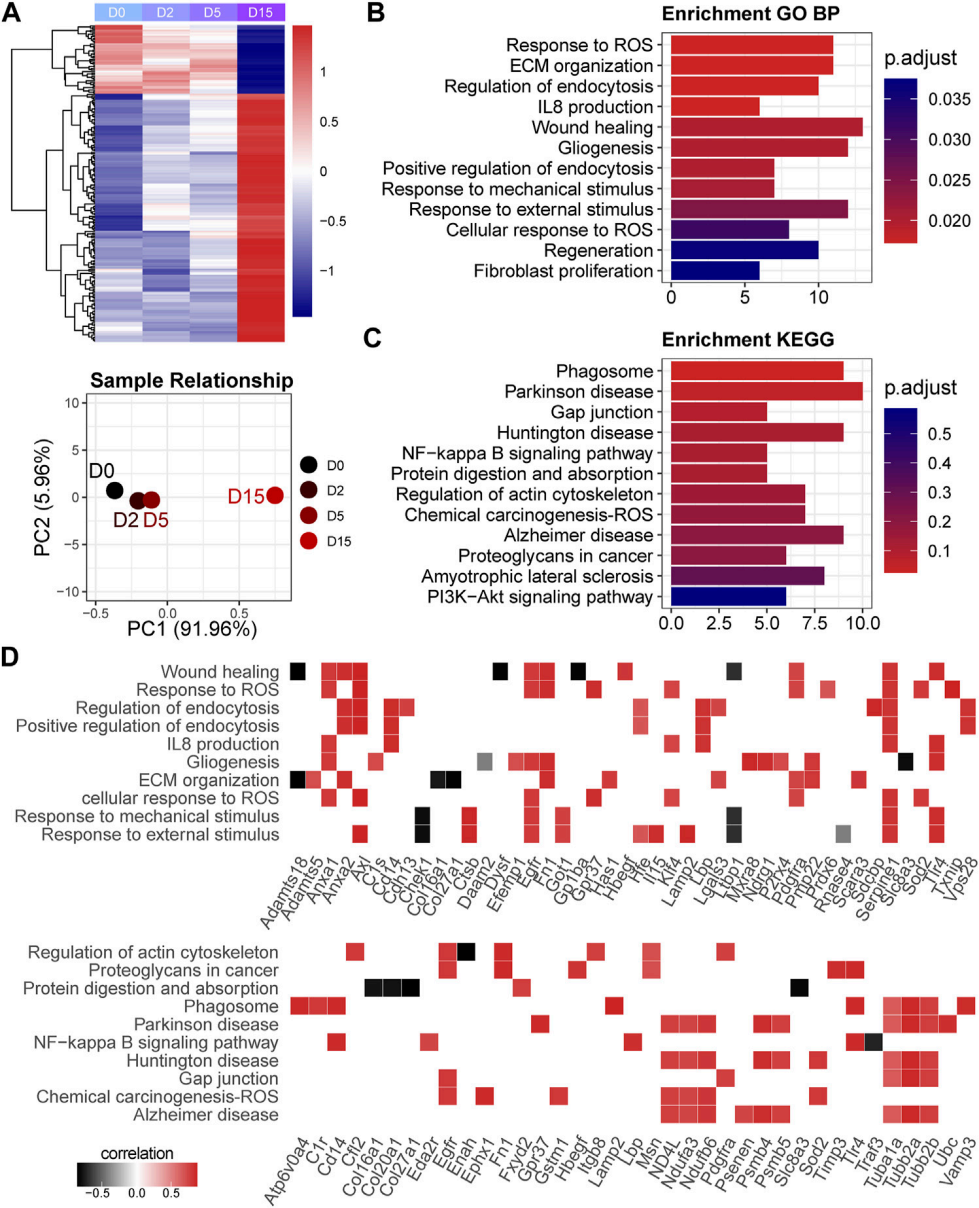
Late responsive genes are related to extracellular matrix organization and cellular stress. **(A)** Heatmap and principal component analysis of late responsive genes (*n* = 518). A notable difference exists between D0 and D15. PC1, the first principal component; PC2, the second principal component. **(B)** GO analysis and **(C)** KEGG pathway analysis of late responsive genes. *p*-value is adjusted by Benjamini and Hochberg method. **(D)** Time correlation of genes in the enriched GO function and KEGG pathways.

A total of 518 genes were included in the M2 module. The heatmap of gene expression and PCA result showed that a notable difference existed between D0 and D15, while a modest difference was found between D0 and D2 or D5 (Figure 5A). As a result, we classified these genes as the late responsive genes in exercise duration. GO analysis showed that these genes were enriched in the biological processes highly related to chondrocyte functions, such as response to ROS, ECM, endocytosis, and interleukin-8 production (Figure 5B). *Prdx6*, *Klf4*, *Pdgfra*, *Sod2*, *Egfr*, *Anxa1*, *Serpine1*, *Gpr37*, *Fn1*, *Axl*, and *Txnip* were enriched in the response to ROS (Supplementary Table S7). KEGG analysis showed that they were related to phagosome, NF-kappa B signaling pathway, and PI3K-Akt signaling pathway (Figure 5C). We plotted the correlations of the enriched genes with time to identify the key genes in these pathways (Figure 5D). Of note, most of the enriched genes were positively correlated with exercise time. *Adamts5*, an OA promoter, was positively correlated with time. Moreover, *Anxa1* which has anti-inflammatory activity showed a positive correlation with time. The genes related to phagosome were positively regulated, indicating an enhanced phagosome function during exercise.

### Time-related signaling pathways are related to metabolism, cell cycle, and immune response

**FIGURE 6:**
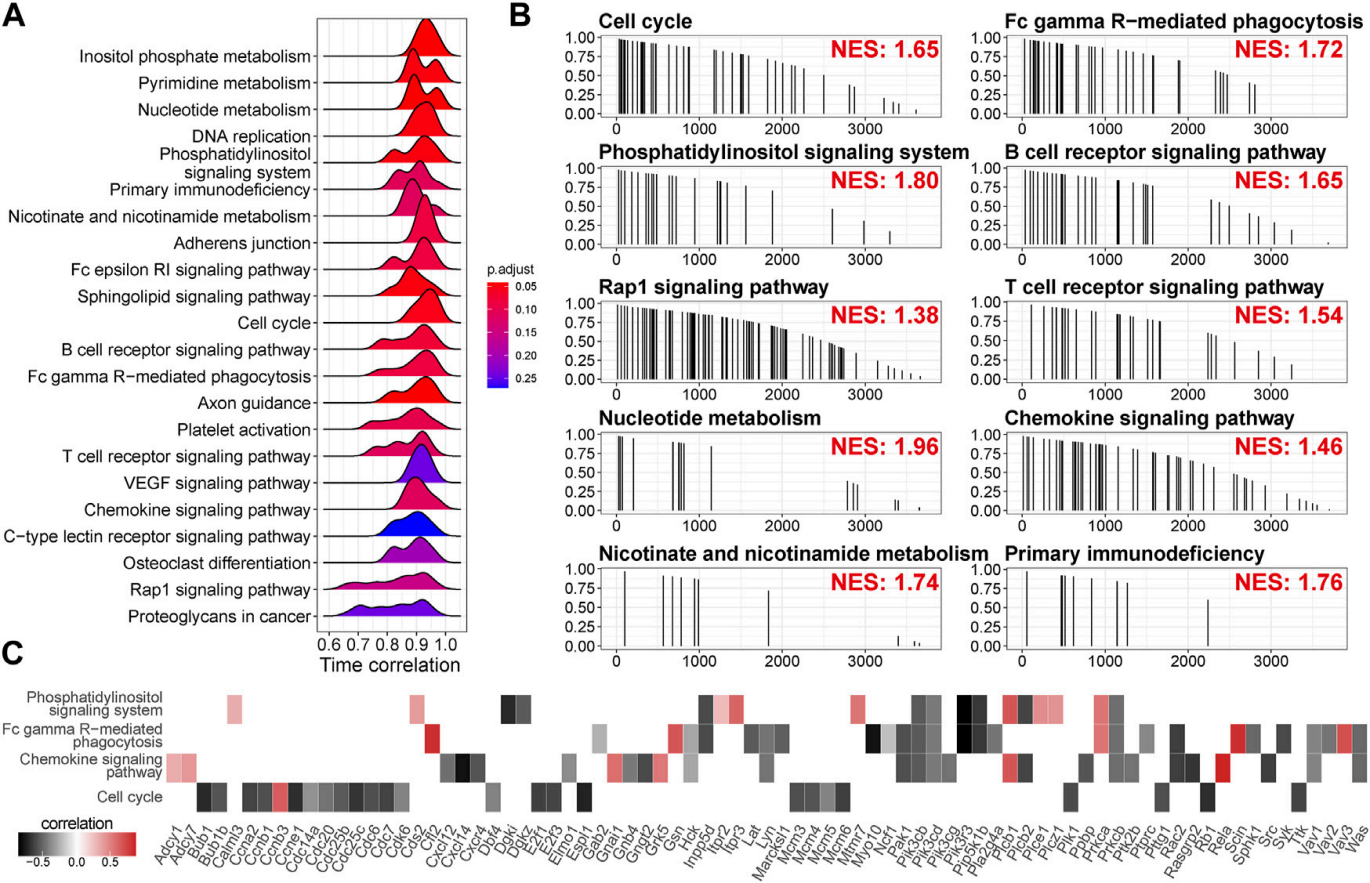
Time-related signaling pathways are related to metabolism, cell cycle, and immune response. **(A)** Time correlation distributions of genes in the individual pathways. The height of peaks indicates the number of the enriched genes. *p*-value is adjusted by Benjamini and Hochberg method. **(B)** Mapping of the genes in the individual pathways along the descending time correlation rank. NES, enrichment score normalized to mean enrichment of random samples of the same size, estimated by gene set enrichment analysis (GSEA). **(C)** Time correlation of the genes in the leading edge of GSEA.

Since we used a strong criterion to select the high time-correlated genes in a module, the gene with a modest time correlation cannot be included. To identify the pathways the highly time-correlated genes are polarized, we employed unbiased mapping without selections. A pre-ranked gene list based on correlation absolution value (0–1) was challenged by serial signaling pathways. As shown in Figures 6A,B, time-correlated genes were polarized in the metabolism pathways (inositol phosphate metabolism, nucleotide metabolism, pyrimidine metabolism, and nicotinate/nicotinamide metabolism), the pathways related to cell proliferation (cell cycle, DNA replication), immune-related pathways (Fc epsilon RI signaling pathway, B cell receptor signaling pathway, T cell receptor signaling pathway, chemokine signaling pathway, Fc gamma R-mediated phagocytosis, and primary immunodeficiency), and other signaling (Rap1 signaling pathway and VEGF signaling pathway). Leading-edge analysis showed the critical enriched genes in individual pathways (Figure 6C). Again, the genes related to cell cycle, such as *Bub1*, *Ccna2*, *Ccnb1*, *Ccne1*, *Cdk6*, *Cdc6*, *Cdc14a*, *Cdc20*, and *Cdc25b*, were negatively correlated with time. Moreover, *Cxcl14* (an immune and inflammatory modulator) and *Pik3r3* (a regulatory subunit of PI3K) showed a strong negative correlation with time.

### Epidermal growth factor receptor is a late responsive gene in the exercised cartilage

**FIGURE 7:**
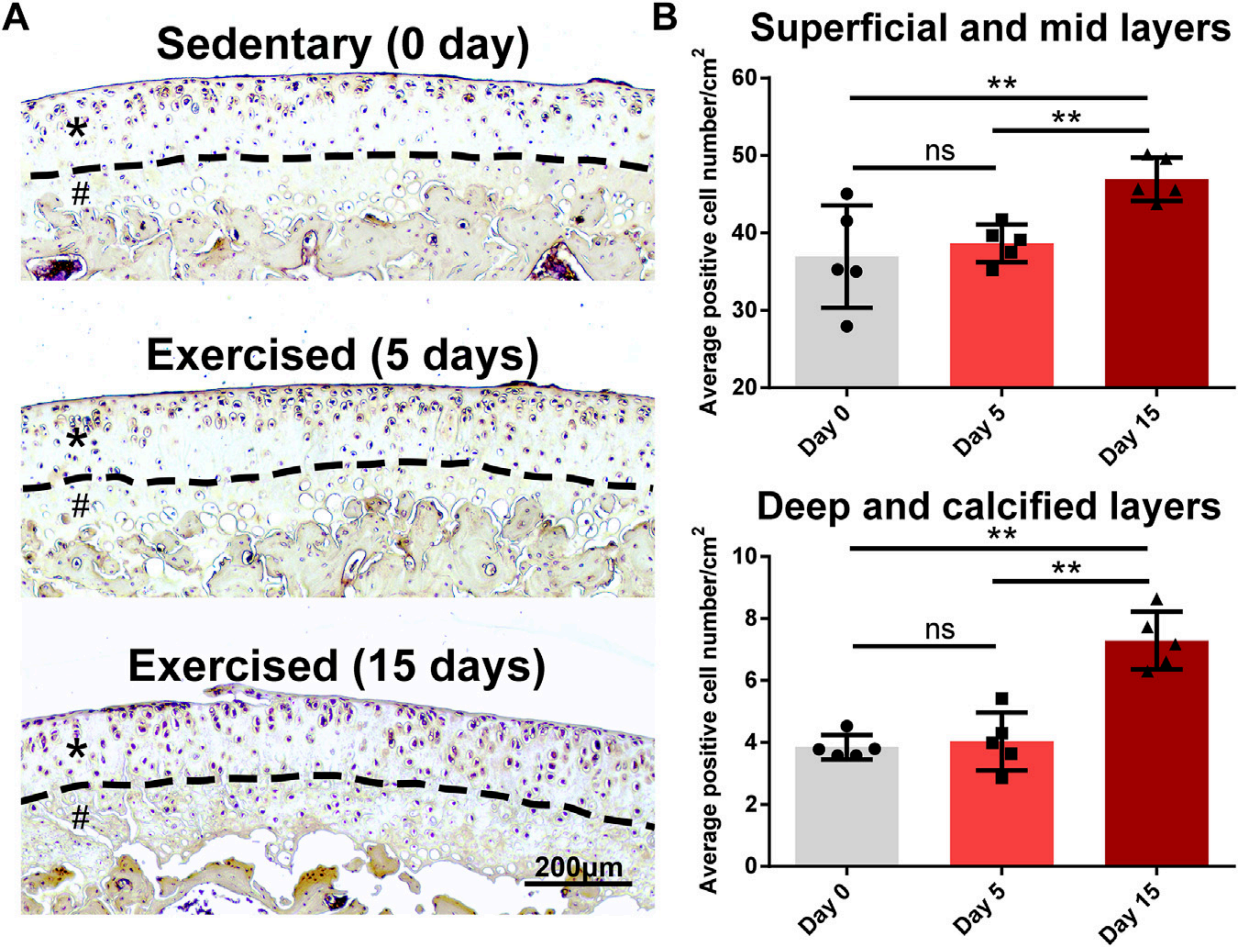
*Egfr* is a late responsive gene in the exercised cartilage. **(A)** *Egfr* expression in the tibial cartilage of the sedentary and exercised rats. *Egfr* is mainly expressed in the superficial layer of rat cartilage. After 15-day exercise, the expression of *Egfr* was increased in the exercised cartilage compared with the sedentary group. Asterisks indicate the superficial and mid layers of cartilage. Hashtags indicate the deep and calcified layers of cartilage. **(B)** The quantitative statistics of the average numbers of EGFR^+^ cells. The EGFR^+^ cells of D15 increased in both surface (superficial/mid) and deep (deep/calcified) layers compared with D0 or D5, while no difference between D0 and D5 was identified. **, *p* < 0.01, One-way ANOVA; ns, not significant.

As we identified Egfr, an important receptor that is a potential target for OA (Wei et al., 2021), as a late responsive gene, we validated its expression in an animal study whose design was based on the previous study (Blazek et al., 2016). As shown in Figure 7A, Egfr was mainly expressed in the superficial layer of rat cartilage. After 15-day exercise, the expression of Egfr was increased in the exercised cartilage compared with the sedentary group (Figure 7A). The quantitative statistics showed that the EGFR + cells of D15 increased in both surface (superficial/mid) and deep (deep/calcified) layers compared with D0 or D5, while no difference between D0 and D5 was identified (Figure 7B).

## Discussion

The favorable outcomes of exercise have been well-documented in OA patients and experimental animal models. However, the heterogeneity of these studies, such as interventions, object characteristics, and outcome definitions, limits the evidence to conclude the benefits of exercise interventions on OA patients and guide the exercise prescriptions (Bartels et al., 2016; Bartholdy et al., 2017; Raposo et al., 2021). So far, the understanding of the effects of exercise dosage is still rare. Blazek et al. (2016) demonstrated the underlying mechanisms of exercise in healthy cartilage by transcriptome analyses. Their findings highlight that exercise is a transcriptional regulator of 147 metabolic pathways, benefiting cartilage by ECM biosynthesis/cartilage strengthening and inflammation suppression. However, the main pathways and key genes related to exercise time have not been fully revealed. The methodological limitation of differential expression analysis is attributed to the fixed threshold, which ignores the mildly-changed genes and gene correlations. In this study, we employed unbiased transcriptomic comparisons to identify the genes and signaling pathways related to exercise or exercise time. More importantly, the time correlation of genes was estimated and two groups of time-correlated genes were identified by modularization. Time-related signaling pathways were also identified by unbiased mapping and polarization of the highly time-correlated genes. Our results suggest that exercise has a notable effect on the cartilage transcriptome. Exercise prominently suppresses the genes related to cell division, hypertrophy, catabolism, inflammation, and immune response, while it shows limited effects on anabolism.

Interestingly, the number of the robustly down-regulated genes (fold-change values < −1 or >1) is ∼2 folds of the numbers of robustly upregulated genes. Moreover, the down-regulated genes are more prominent and stable over time than the upregulated genes. These findings indicate that exercise trends to suppress gene expression in such an exercise design. It seems that exercise benefits cartilage in the strategy of inhibiting inflammation-related events and elevating metabolism at the expense of cell cycle blockage. In the prominent responsive gene panels, *Hist1h1a*, coding H1.1 linker histone, is a basic nuclear protein responsible for nucleosome structure of the chromosomal fiber, modulating the accessibility of regulatory proteins, chromatin-remodeling factors, and histone modification enzymes to their target sites (Hergeth and Schneider, 2015). Beside for this gene, a batch of histone genes, such as *Hist1h1b*, *Hist1h2ail*, *Hist1h2ao*, and *Hist1h2ba* (Supplementary Table S2), were also suppressed by exercise. These genes may be attributed to the prominent gene suppression induced by exercise while the role of these genes has not been documented. *Stk32b* is a serine-threonine kinase that belongs to the calcium/calmodulin-dependent family of kinases (Mujica et al., 2001). *Stk32b* is characterized as an oncogene activating the PI3K-AKT pathway (Zhou et al., 2020). Again, no evidence has demonstrated the role of *Stk32b* in cartilage. Since the PI3K-AKT pathway is also identified in our data, *Stk32b* may regulate this pathway as a response to exercise.

Although exercise time does not prominently contribute to the effects of exercise, it is a factor related to a batch of cellular functions and signaling pathways that are critical for chondrocyte biology and OA development, such as ECM homeostasis and cellular response to growth factors and stress. The transcriptomic remodeling over time is for the first time characterized by an unbiased method. Two clusters of genes were identified according to the expression pattern over time. The early responsive genes are of great interest because they are also in high relation to the overall effects of exercise. It is speculated that the main effects of exercise on cartilage transcriptome may start to occur in an early period of exercise because the transcriptome of cartilage is sensitive, at least, to mechanical loading.

TGFβ/BMP signaling pathway is critical for cartilage homeostasis (Wang et al., 2014; Thielen et al., 2019). Importantly, BMP signaling is identified as early responsive to exercise in this study. The expression of Cav1, Sfrp5, and Tgfbr3 are positively related with exercise time. Caveolin-1 (*Cav1*), the signature structural protein of caveolar membrane, is well-documented in cellular stress, senescence, and aging (Ha et al., 2021; Gokani and Bhatt, 2022). As a regulator of signaling events, *Cav1* modifies the response to BMPs by the interaction with BMPRII (Wertz and Bauer, 2008; Boscher and Nabi, 2012; Tomita et al., 2021). In OA articular cartilage, *Cav1* expression is higher than the normal control and is positivity was associated with cartilage degeneration (Dai et al., 2006; Min et al., 2015). Catabolic stress induced by IL-1β or H_2_O_2_ promotes chondrocyte senescence and catabolism via upregulation of *Cav1* (Dai et al., 2006). The expression of *Cav1* can in turn modify the cellular response to stress. *Cav1* represses the activation of unfolded protein response, attenuating PERK/IRE1α signaling and increasing susceptibility to ER stress and hypoxia in cancer cells (Diaz et al., 2020). Moreover, *Cav1* can modify autophagy. *Cav1* deficiency promotes autophagy *via* enhancing lysosomal function and autophagosome-lysosome fusion (Shi et al., 2015). In our study, the positive regulation of exercise time on *Cav1* expression may indicate that exercise induces cellular stress in chondrocytes and *Cav1* may regulate the cellular response to stress by modifying autophagy. Moreover, *Cav1* is a negative regulator of MMP-1 expression via inhibition of Erk1/2/Ets1 signaling pathway (Haines et al., 2011). Upregulation of *Cav1* by exercise may inhibit the expression of MMPs in chondrocytes, which may explain the downregulation of MMPs found in our study. *Sfrp5* functions as positive regulators of BMP signaling (Meyers et al., 2013; Holly et al., 2014). Moreover, *Ppp2r1b*, *Prkaa2*, and *Sgk1* in the PI3K-Akt signaling also showed a positive correlation with time. The PI3K-Akt signaling pathway is essential for cartilage maintenance and OA pathogenesis (Sun et al., 2020). Treadmill exercise on animal models attenuates OA by modifying the PI3K/AKT pathway (Lu et al., 2021; Jia et al., 2022).

EGFR is a trans-membrane receptor tyrosine kinase (Dutta and Maity, 2007). EGFR signaling is critical for maintaining adult cartilage homeostasis and attenuating osteoarthritis progression (Jia et al., 2016; Mchugh, 2021; Wei et al., 2022). Genetic or pharmacologic activation of EGFR attenuates surgery-induced OA cartilage degeneration, subchondral bone plate sclerosis, and joint pain (Wei et al., 2021). As the expression of Egfr was increased by long-term exercise in our study, long-term exercise may enhance EGFR signaling by upregulating the receptor expression, which could protect the cartilage from osteoarthritis. However, it is possible that the upregulation of Egfr is the feedback of the suppression of EGFR signaling pathway, which may suggest that the exercise intensity of 15 days would attenuate this pathway and induce osteoarthritis. Moreover, accumulating evidence has revealed the roles of tyrosine kinases in signaling pathways promoting chondrocyte hypertrophy, highlighting their potential as therapeutic targets for osteoarthritis (Ferrao Blanco et al., 2021). EGFR signaling is also thought to drive ECM degradation through the regulation of MMP-9 and MMP-13 (Zhang et al., 2013). The relationship between EGFR signaling and chondrocyte hypertrophy/cartilage degradation in exercise needs to be considered in the future. The paradox of EGFR signaling in osteoarthritis may be explained by the dynamic function of this pathway during osteoarthritis progression.

Although this study reveals the potential mechanisms of exercise time, several limitations need to be considered. First, small sample size may harm the reliability of the results and conclusions. Secondly, we only considered one specific exercise model in a limited time period. Moreover, the effects of gender and age were not considered in this study. Finally, the detailed functions of genes and pathways in exercised cartilage were not demonstrated although we confirmed the expression of specific interested genes by experimental validations.

## Conclusion

This study elucidated the effects of exercise on cartilage transcriptome and identified time-related transcriptomic reprogramming by unbiased comparisons and modularization methods. Early and late responsive genes are related to cartilage homeostasis and OA development. Time-related signaling pathways are related to cellular metabolism, cell cycle, and immune response.

## Data Availability

Publicly available datasets were analyzed in this study. This data can be found here: https://www.ncbi.nlm.nih.gov/geo/query/acc.cgi?acc=GSE74898.

## Supplementary material

SUPPLEMENTARY FIGURE S1 Common differentially expressed genes of the comparisons of the exercised and control groups and the long-time and short-time groups.

SUPPLEMENTARY TABLE S1 The differentially expressed genes of the comparisons between individual time points.

SUPPLEMENTARY TABLE S2 The differentially expressed genes of the comparisons of the exercised and control groups.

SUPPLEMENTARY TABLE S3 GO and KEGG analysis of persistent upregulated genes.

SUPPLEMENTARY TABLE S4 GO and KEGG analysis of persistent downregulated genes.

SUPPLEMENTARY TABLE S5 The correlation of genes in M1 and M2 modules with exercise time or the eigengene of the module.

SUPPLEMENTARY TABLE S6 GO and KEGG analysis of early responsive genes.

SUPPLEMENTARY TABLE S7 GO and KEGG analysis of late responsive genes.
