# Supplementary figures and images for: Unbiased comparison and modularization identify time-related transcriptomic reprogramming in exercised rat cartilage: Integrated data mining and experimental validation
Source: Front Physiol. 2022 Sep 15;13:974266. doi: 10.3389/fphys.2022.974266 (PMC9520919; doi:10.3389/fphys.2022.974266)

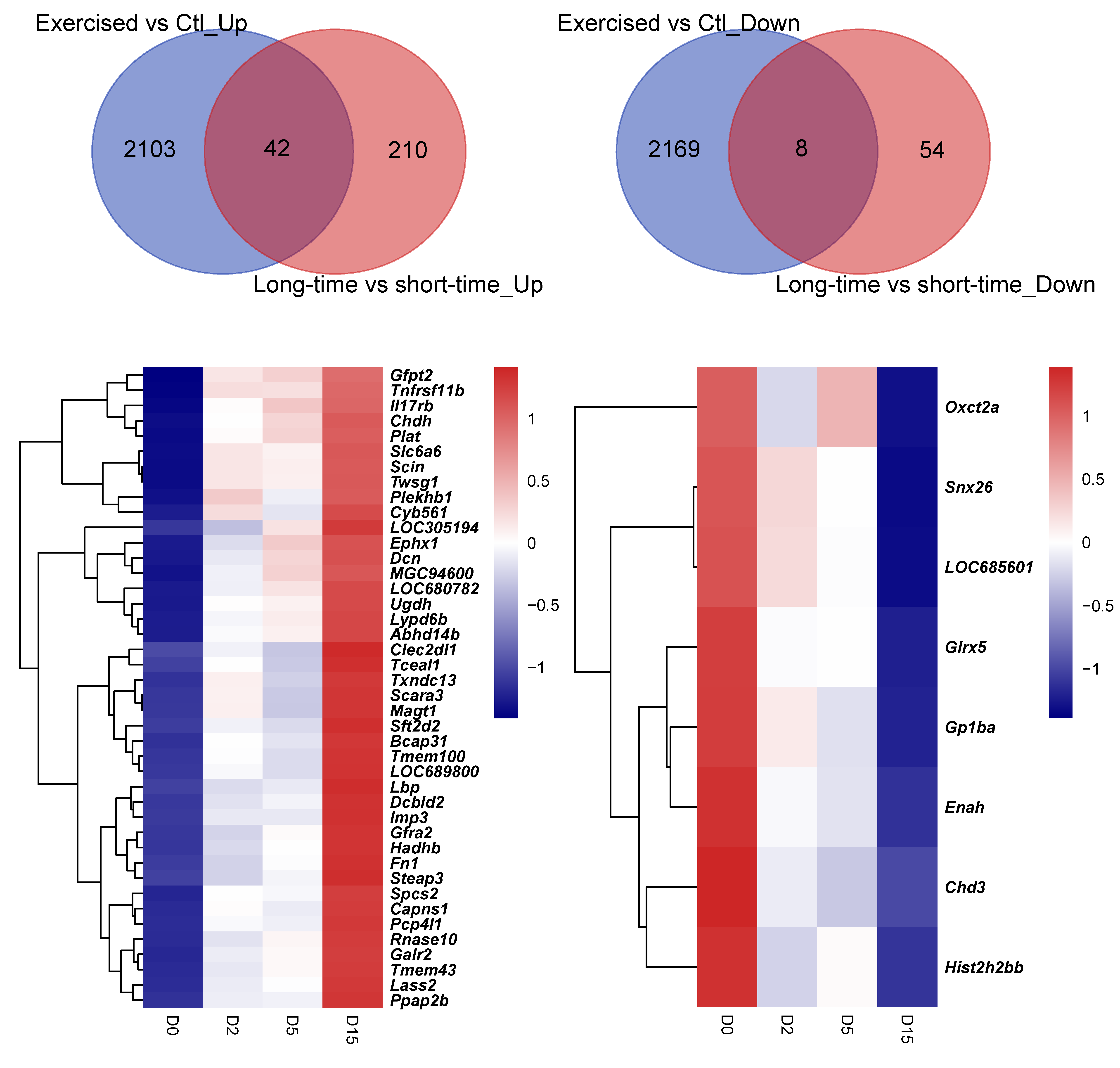

Supplement: Supplementary file 3 [file Image1.TIF]
